# Supplementary material for: Are carbon emissions trading and green financial instruments synergistic? -Comprehensive quantitative research based on content analysis
Source: PLoS One. 2024 Mar 7;19(3):e0298601. doi: 10.1371/journal.pone.0298601 (PMC10919601; doi:10.1371/journal.pone.0298601)
Supplement: S1 Data — (ZIP) [file pone.0298601.s001.zip › Data/地级市Z_F工作报告.zip!/╝¬┴╓/│ñ┤║╩╨/└┤╫╘╣1⁄2╓┌║┼í╛┬φ┐╦╩2╛▌═°í┐_2016.docx]

各位代表：

现在，我代表市政府，向大会作政府工作报告，请予审议，并请市政协委员提出意见。

一、“十二五”时期工作回顾

“十二五”时期，是长春发展进程中极不平凡的五年。在省委、省政府和市委的正确领导下，在人大、政协的监督支持下，我们紧紧依靠和团结全市人民，主动适应经济发展新常态，从容应对各种风险挑战，推动老工业基地振兴取得了新的历史性进步，建设幸福长春取得了新的可喜成就。

——这是经济发展实力稳步增强的五年。全市经济总量从3329亿元增加到5530亿元，年均增长9.3%。地方级财政收入从180.8亿元增加到388.2亿元，增加1.15倍。粮食产量实现“十二连丰”，传统制造业加快转型升级，现代服务业翻番式增长，新产业、新业态、新技术、新商业模式不断涌现。民营经济主营收入突破1万亿元，发展活力和贡献率显著提高。

——这是城市建设质量不断提升的五年。新的城市次中心在绕城高速公路以外竞相成长，主城区700多万平方米棚户区、4000多万平方米老旧楼宇经过改造旧貌换新颜。基础设施建设扎实推进，适应百万辆机动车保有量的立体交通体系基本形成，水、气、热供给能力实现从紧平衡到相对宽松的转变。

——这是人民生活面貌持续改善的五年。教育、文化、卫生、体育事业全面进步。城镇居民人均可支配收入年均增长11.1%，快于经济增长。农村居民人均可支配收入年均增长11.7%，快于城镇居民收入增长。城乡养老、医疗保险覆盖率均达到95%以上，初步实现人人享有基本社会保障。长春连续当选最具幸福感城市。

这份沉甸甸的成绩单为“十二五”画上了圆满的句号，凝聚着全市上下的共同努力和心血汗水，展示了广大干部群众建设幸福长春的坚定决心和必胜信念。

五年来，主要做了以下工作：

（一）始终把扩大有效投资作为第一抓手，推动经济在新常态下转型升级。我们坚持把项目建设摆在经济工作首位，用增量改造传统动能、培育发展新动能，努力推动经济实现更高质量、更有效率、更可持续的发展。五年累计实施亿元以上项目2650个，完成固定资产投资17339亿元，分别相当于“十一五”的2倍和1.8倍。

传统产业在规模扩张中优化升级。整车产量由160万辆提高到220万辆，轨道客车修造量由2000辆提高到4000辆，农产品加工业产值增加1.2倍，三大主导产业新产品产值率达到76%。体现工业质量效益的工业增加值率由22.8%提高到25.3%。一批重大农业增产技术得到推广，粮食亩产由887.2斤提高到1066.4斤，粮食产量达到191.1亿斤。长春被命名为“中国优质粳米之都”。综合农机化水平由64.8%提高到78.5%。家庭农场、农民合作社等新型经营主体数量增加8.5倍。畜禽规模饲养比例达到80%。积极培育文化、养老等服务性消费，社会消费品零售总额增长87%。旅游业总收入突破千亿元大关。启动建设41个商业综合体，300万平方米商务面积投入使用。引入29家域外金融机构，区域性金融服务能力显著提升。2户企业上市，22户企业新三板挂牌。服务业投资连续保持高速增长，对经济增长贡献率提高17.4个百分点。

新兴产业成为支撑增长重要力量。先进装备制造、光电信息、生物制造、医药健康、新能源及新能源汽车、新材料产业投资额增长2.8倍，产值占规模以上工业产值比重从9.4%提高到17.1%。文化创意、信息服务等现代服务业集聚区入驻企业达到2250户。健康休闲、物流快递等新兴业态竞相成长。服务外包执行金额增加1.4倍。大力发展“互联网+”，电子商务企业超过200户、个体网店数量超过3万个，近1/4规模以上工业企业、1/3限额以上商业企业发展网上业务。成功发射“吉林一号”商业卫星，聚乳酸、无人机、工业机器人、单克隆抗体等一批高科技成果实现转化。科技金融创新服务中心投入运行。建成摆渡创新工场等10个众创空间，科技企业孵化器面积达到180万平方米，在孵科技企业超过千户。国家高新技术企业、科技型“小巨人”企业分别达到242户和400户，科技创新驱动发展能力不断增强。

（二）始终把全面深化改革作为第一动力，持续激发和释放发展新动能。我们坚持把全面深化改革作为头等大事，突出抓好具有标志性、关联性作用的重大改革举措落地，努力向改革要动力、要潜力、要红利。

扎实推进政府自身改革。取消下放577项审批权限，减放比例达到70.1%，市本级非行政许可实现“零审批”。撤销89个议事协调机构。事业单位改革稳步实施。改进行政审批服务。深化商事制度改革，降低市场准入门槛。不断完善民营经济融资担保、公共技术、要素配置等公共服务平台。政府权力的减法、服务的加法换来了市场活力的增强，五年累计新登记私营企业5.5万户，大众创业、万众创新热潮初步形成。

全力实施重点领域改革。新型城镇化、服务业综合改革、水生态文明建设、民营经济综合配套改革、农村集体产权制度改革、养老服务业综合改革等试点顺利实施。行政事业单位国有资产管理改革、集体林权制度配套改革完成阶段性任务。政府购买公共服务改革稳步推进。国有企业改革取得新进展。全面规范义务教育阶段公参民学校办学行为。基本完成文艺院团转企改制。市级以下公立医院启动综合改革。

坚持以对外开放促改革。主动融入“一带一路”战略，深度推进长吉图开发开放。兴隆综合保税区封关运行。一汽、长客等重点企业走出去到海外建立生产基地。皓月、希达等一批企业到境外设立产品展示营销中心。汽车零部件等高附加值机电产品出口额实现翻番。在长投资世界500强企业达到70户，五年实际利用外资增长2倍。

（三）始终把绿色宜居城市作为第一任务，稳步推进新型城镇化建设。我们以绿色、智慧、人文理念为指导，启动实施了新一轮的城市大建设、大改造。五年累计完成城建投资1625.1亿元，相当于“十一五”的2.65倍。

优化城市空间布局。主城区通过搬迁工业企业、拆除棚户区、完善基础设施，加速实现产业、功能和环境的有机更新。长东北、莲花山、经开保税等产业园区多点发力、齐头并进，汽开、净月、高新南区竞相建设、加速成长，九台卡伦、德惠米沙子、农安合隆、双阳奢岭积极承接主城区带动和产业转移。旧城与新城、城市与县域良性互动，重构了城市框架，优化了功能布局，也打开了长春未来的发展空间。

提升城市承载能力。“两横三纵”快速路投入使用，四环以内主要道路完成大中修。启动建设地铁1号线、2号线、北湖快轨，轻轨4号线投入运营。城市日供水能力由86万吨提高到116万吨，增加35%。天然气日供给能力由132万立方米提高到227万立方米，增加72%。供热能力由1.01亿平方米提高到1.89亿平方米，增加87%。污水日处理能力由85.8万吨提高到172.8万吨，增加1倍。新建改造各类地下管网5099公里，自来水漏失率下降17%，供热管网完好率达到89%，燃气管网高危率降低到5%以下。主城区清扫保洁面积由4305万平方米增加到6850万平方米，机械化清扫率达到73%。

改善城市环境质量。新建大块绿地229块。主城区对外开放公园数量增加1倍以上。伊通河综合整治取得实质性进展。淘汰3.2万辆黄标车，拆除1828台燃煤小锅炉，62.5万户煤制气居民用户完成天然气置换，狠抓工业烟粉尘治理、秸秆综合利用，PM10、PM2.5自2013年达到峰值以后分别下降17.8%和10.9%。修复文庙、杂技宫、沙俄领事馆等一批历史文化建筑。长影老厂区等18项文物晋级全国重点文物保护单位。净月潭、长影世纪城晋级国家5A级旅游景区。持续开展市容环境综合整治，从严整治管理顽疾。深入开展文明城创建，长春进入全国文明城行列。

（四）始终把保障改善民生作为第一目标，努力让广大群众有更多的获得感。我们坚持每年把新增财力70%以上用于改善民生，连续实施5个年度行动计划，为人民群众办了390余件实事。

提高居民收入。积极促进就业，城镇累计新增就业62.9万人，累计安置下岗失业人员29.2万人，城镇登记失业率始终控制在4%以下，“零就业家庭”保持动态为零。4次提高职工最低工资标准。在岗职工平均工资提高56.8%。城镇职工医疗保险、城镇居民医疗保险、新农合参保率均接近100%，城镇职工基本养老保险参保率达到95.5%，城乡居民养老保险参保率均超过95%。职工养老金、居民养老金、失业保险金标准分别提高64.5%、127%、151%。

改进公共服务。义务阶段公办学校全部就近划片入学，创建新优质学校127所，城区通过国家义务教育基本均衡评估验收。完成国家公共文化服务体系示范区创建，区（县）图书馆、乡镇文化馆、村文化室全部达标并免费开放。社区、行政村体育设施配置率分别达到100%和60%。县医院、乡镇卫生院、村卫生室全部完成标准化建设。养老机构床位数量增长1.84倍，居家、社区养老服务新模式在全国推广。深入开展幸福社区创建。主城区60%以上的社区用房建筑面积达到1000平方米。

扶助困难群众。5次提高城乡最低生活保障标准，城镇低保家庭老、少、病、残人员等特殊群体补助标准再增加50%。建立城乡居民临时救助、特困群众参加基本养老保险扶助制度。城区困难群众患急难重症到定点医疗机构“先住院、后付费”。残疾儿童抢救性康复救助、贫困重性精神病患者康复救助、贫困白内障患者复明手术实现“有一助一”。困难群众法律援助基本做到应援尽援。累计建设3.2万套保障性住房，为5.6万户居民发放租房补贴，低收入困难群众住房保障基本实现应保尽保。

改善人居环境。基本消除农村泥草房、城市D级危房。综合整治230个老旧散小区。主城区1007座二次供水泵站完成改造。158万农村居民饮水安全问题得到解决。三环以内巷道基本完成改造。创建公交都市示范城市，开辟优化60条公交线路，累计更新公交车2500余台。施划机动车停车泊位3.8万个。一批人行过街天桥投入使用。新建农村公路5998公里、桥梁280座。

强化社会治理。积极构建“人性化、网格化、信息化、法治化”社会治理模式，全面完善社会治安防控体系，城区重点区域高清探头实现全覆盖，公安干警对城区实施24小时不间断巡防，110警情、“两抢”案件、命案数量连续5年下降，长春荣获全国社会管理综合治理最高荣誉“长安杯”。深入开展国家食品安全城市创建试点，切实提升食品药品安全监管能力。全面抓好防震减灾，长春在全国考核评比中荣膺榜首。狠抓安全生产，累计排查整改安全隐患23.1万项，安全生产事故总数下降53.5%。

（五）始终把加强依法行政作为第一要求，加快建设服务型政府。我们始终坚持全心全意为人民服务的根本宗旨，不断加强政府自身建设，努力为群众提供优质高效服务。

主动接受监督。自觉接受人大及其常委会的法律监督、工作监督和政协的民主监督，坚持重大事项向人大报告、与政协协商制度。认真听取各民主党派、工商联以及无党派人士的意见建议。办理人大议案19件、人大代表建议1022件、政协建议案15件、政协提案1537件。

强化依法行政。制定政府部门权力清单、责任清单，认真执行规范性文件合法性审查、行政决策合法性分析及社会稳定风险评估机制，严格约束行政处罚自由裁量权，全面实施行政复议案件集中办理、依法行政示范单位创建，不断提升规范公正文明执法水平。

切实转变作风。深入开展党的群众路线教育实践活动、“三严三实”专题教育，大力整治庸政、懒政、怠政。坚决执行中央八项规定，停建楼堂馆所，清理公车、办公用房，实施公务卡制度，严格控制“三公”经费支出。

加强廉政建设。严格落实廉政建设主体责任和监督责任，着力健全惩治和预防腐败体系，及时公开市本级公共财政收支预算、市直部门“三公”经费预算决算，加大审计监督力度，强化项目招标、政府采购等重点领域监管，始终保持惩治腐败的高压态势。

努力化解矛盾。建成市民来信来访集中受理大厅，全面提高市长公开电话、局长接待日、读报读网工作水平，完善多元化矛盾纠纷化解机制，切实解决群众反映强烈的信访突出问题，群众信访总量稳步下降。

隆重纪念中国人民抗日战争暨世界反法西斯战争胜利70周年。军政军民团结奋进，创建全国双拥模范城获得“七连冠”。

尤其是2015年，面对“十二五”收官的决战之期、复杂严峻的发展环境，全市上下坚持稳中求进工作总基调，积极适应经济发展新常态，团结一心、善为实干、攻坚克难，推动振兴发展取得了新的突破，为“十三五”发展奠定了坚实基础。

一是经济在应对下行中保持稳定增长。先后出台17项稳增长政策，接连采取结构性减税等一揽子措施，全面加大对重点企业帮扶力度，全市经济止跌回稳、逐步提速，增长速度由一季度的5.9%、二季度的6.1%、三季度的6.2%，逐步回升到全年的6.5%，重新回到经济运行合理区间。

二是产业在提质增效中加快转型升级。战略性新兴产业迅速壮大，产值增速超过规模以上工业17.5个百分点。服务业明显提速，占经济比重提高2.56个百分点。“互联网+”行动计划稳步实施，电子商务交易额增长23%，众创、众包、众扶、众筹等新兴业态日渐兴起，新的增长动力加快形成并不断蓄积力量。

三是市场在改革开放中迸发内在活力。“五证合一”登记等37项重点改革任务取得突破。削减了53.9%的政府部门行政职权，行政审批时限整体压缩31%。“一门式”公共服务改革试点取得成效。民营经济综合配套改革向纵深推进，新登记私营企业数量增长21%。“长满欧”国际货运班列、长春至莫斯科货运航线正式开通，跨境电子商务出口业务量跃居全国第三。

四是城市在完善功能中提升质量档次。主城区交通指挥系统完成升级改造，高峰期主干路车速提高15%。国家西气东输干线、长春外环高压管网投入使用，冬季“气荒”全面缓解。百里伊通河生态长廊、百公里地下综合管廊工程全面启动。基本完成16个城市出入口改造。城区新增绿化面积相当于上年的1.9倍。

五是民生在保障兜底中惠及更多群众。城镇新增就业12.6万人，2万套公租房分配到户，7个城区托老中心、120个社区居家养老服务中心投入使用，失能人员医疗照护保险制度全面推行，农村贫困人口减少1.7万人，在经济下行压力不断加大的情况下民生状况得到新的改善和提升。

各位代表，过去五年经济社会发展取得的重大成就，源自于省委、省政府和市委的正确领导，得益于人大、政协以及社会各界的大力支持，归功于全市广大干部群众的团结奋斗。在此，我代表市政府，向为长春发展做出突出贡献的全市人民致以崇高的敬意！向给予我们监督与支持的人大代表、政协委员和社会各界人士，向积极参与长春发展建设的中省直驻长单位，人民解放军和武警驻长部队以及海内外的朋友们，表示衷心的感谢！

成绩来之不易、经验弥足珍贵。“十二五”发展实践充分说明：必须把发展作为解决长春所有问题的关键，始终坚持加快发展不动摇，努力实现更高质量、更有效率、更加公平、更可持续的发展；必须把改革开放作为推动长春发展的根本动力，不断解放思想、不断解放和发展社会生产力、不断解放和增强社会活力；必须把创新摆在长春发展振兴的核心位置，深入落实创新驱动发展战略，全面推动大众创业、万众创新，努力培育形成新的增长动力源泉；必须把改善民生、增进人民福祉作为发展的根本目的，多谋民生之利、多解民生之忧，真正把发展成果体现到人民生活改善上；必须把法治政府建设作为发展的重要保障，严格依法行政、深入简政放权、加快职能转变，努力以政府权力的减法换市场活力的加法。这些经验体会，是我们在实践中积累的宝贵财富，必须继续坚持和发扬。

在肯定成绩的同时也要清醒看到，前进的道路上还面临不少困难和问题。经济下行压力有增无减，结构调整、转型升级任务繁重；创新创业氛围不足，内生动力活力尚需释放；环境污染问题日益显性化，雾霾、水体治理任重道远；就业结构性矛盾突出，住房、医疗、养老、教育、收入分配、征地拆迁等方面群众不满意的问题依然较多；安全基础设施不完善，安全事故高发势头还没有从根本上得到遏制；政府依法行政水平不高，不廉不勤现象仍然存在。对于这些问题，我们必须高度重视，并在今后工作中认真加以解决。

二、“十三五”时期主要任务

“十三五”时期，是全面建成小康社会的决胜期、推动振兴发展的关键期。我们既要深刻认识形势的严峻性、复杂性和紧迫性，直面经济增速换挡、结构调整阵痛、动能转换困难叠加交织带来的艰巨挑战。更要清醒地看到，长春经济韧性好、潜力足、回旋余地大的基本面没有变，经济持续增长的良好支撑基础和条件没有变，结构调整优化升级的前进态势没有变，特别是全市广大干部群众满怀发展激情、奋发有为的精神力量势不可挡。只要我们坚定信心、群策群力、埋头苦干，就一定能够闯关夺隘、奋勇向前、行稳致远，把长春发展推向新的阶段。

今后五年，政府工作的指导思想是：深入贯彻党的十八大，十八届三中、四中、五中全会精神，认真落实习近平总书记系列重要讲话特别是视察吉林重要讲话精神，按照省委十届六次全会和市委十二届八次全会部署，以“四个全面”战略布局为引领，以新一轮振兴发展为主题，以建设吉林中部创新转型核心区为重点，坚持变中求新、变中求进、变中突破，发挥“五个优势”、推进“五项举措”、加快“五大发展”，努力走出一条质量更高、效益更好、结构更优、优势充分释放的发展新路，奋力夺取全面建成小康社会的伟大胜利，努力在实现“中国梦”的伟大征程中追逐“长春梦”。

今后五年，必须牢固树立并切实贯彻创新、协调、绿色、开放、共享的发展理念。坚持创新发展，把创新摆在发展全局的核心位置，让创新贯穿经济社会发展全过程，让创新在全社会蔚然成风。坚持协调发展，牢牢把握全面建成小康社会任务，努力拉长“短板”，不断提高发展的协调性和平衡性。坚持绿色发展，严格环境保护、强化生态修复、节约利用资源，努力建设美丽长春。坚持开放发展，着力引进域内外人才、资金、技术，搭建开放平台，畅通开放渠道，不断提高开放水平。坚持共享发展，更加注重保障和改善民生，使全体人民在共建共享中有更多获得感，朝着共同富裕方向稳步前进。

今后五年，全市经济社会发展的目标任务是：

——经济实力更强。我们要更加重视质量、更加重视效益、更加注重科技创新，在不断转变发展方式、优化经济结构、提高发展可持续性的基础上，确保GDP保持7%左右的中高速增长，财政收入增幅与经济增长相适应，综合实力进入中等发达城市行列。

——发展动能更高。我们要充分发挥创新对发展的支撑引领作用，加快培育新产业、新业态、新技术、新商业模式，推动发展新动能不断壮大。全面实施传统产业升级改造，不断提升产业层次、素质和竞争力，让传统产业持续焕发生机和活力。

——经济结构更优。我们要调整产业结构，农业要争当现代化排头兵，制造业要迈向中高端，战略性新兴产业要在全国形成较强竞争力，服务业占经济比重要逐年提高。调整增长结构，发挥有效投资关键作用的同时，大幅提高消费的经济增长贡献率。

——城市面貌更新。我们要高质量建设新城，高水平改造旧城。落实公交优先战略，基本确立现代综合交通体系。基本完成棚户区、老旧楼宇“暖房子”、历史文化街区改造，切实加强城市管理，全面优化城市形象。大力发展城市文化，努力培育城市精神，促进软实力与硬实力同步提升。

——区域发展更快。我们要坚定不移地支持开发区转型升级，加速成为承载新型工业化、高端服务业的新城区。城区要成为服务业主战场，在环境建设、民生改善、社会和谐等领域实现全面进步。县（市）都要成为中等城市，人口加速向城镇集聚，新农村建设全面推进。

——改革开放更广。我们要全面深化重要领域改革，坚决破除一切不合时宜的束缚和桎梏，充分释放改革红利、动力和潜力。更加积极主动地对外开放，主动融入“一带一路”战略，深入实施长吉图开发开放，全面提升开放型经济水平。

——生态环境更好。我们要不断加大自然生态系统修复保护力度，完成百里伊通河生态长廊综合改造，新建改造一批城市公园、绿地。综合治理大气和水体污染，积极推行循环经济、低碳经济等绿色经济模式，努力把长春建设成为天蓝水净、空气清新的绿色宜居森林城。

——社会大局更稳。我们要加强基层社会管理和服务体系建设，充分发挥群众参与社会管理的基础作用。完善重大决策社会稳定风险评估机制，从源头减少和化解社会矛盾。健全民主法制，保障公共安全，努力开创社会和谐人人有责、和谐社会人人共享的生动局面。

——民生福祉更多。我们要加快完善基本公共服务体系，在学有所教、劳有所得、病有所医、老有所养、住有所居上持续取得新进展。大力推动富民增收，群众收入差距不断缩小，中等收入群体持续扩大，农村贫困人口全部脱贫，城镇低保群众得到更有针对性的帮扶，全市人民共同迈入全面小康社会。

根据上述考虑和安排，市政府制订了《长春市国民经济和社会发展第十三个五年规划纲要(草案)》，并经市委十二届八次全会讨论决定提交本次人代会审议。

三、2016年重点工作

今年是“十三五”开局之年。面对依然复杂严峻的外部宏观环境和交织叠加的各种矛盾困难，我们既要积极适应、把握、引领新常态，在“变”中保持定力、趟出新路，更要认清、抢抓、用好新机遇，在“不变”中坚定信心、推动发展，以充分的准备、扎实的努力去争取最好的结果，确保“十三五”实现开门红。

全市经济社会发展主要预期目标是：地区生产总值增长7%左右，城镇居民人均可支配收入和农村居民人均可支配收入与经济基本保持同步增长，万元GDP能耗降低3%，城镇登记失业率控制在4%以内。

实际工作中，我们不仅要保持经济持续稳定增长的好势头，更要在解放思想中凝聚发展“新气场”，在改革创新中培育发展“新动力”，在转型升级中构建发展“新优势”，在改善民生中定位发展“新坐标”，确保各项工作取得新的更大成效。

（一）保持经济稳定增长

把稳增长摆在突出位置，挖掘增长潜力、激发市场动力、释放企业活力，确保经济运行在合理区间。

统筹三次产业支撑增长。对产值超10亿元工业企业跟踪包保，稳定经济增长大局。积极培育工业增长点，新增规模以上企业200户。促进健康、旅游、文化、绿色、养老消费，扩大移动互联网等信息消费。加快房地产去库存，稳定住房等大宗消费。社会消费品零售总额增长8%左右。夯实农业稳产增产基础，粮食产量正常年景下不低于180亿斤。

扩大投资规模拉动增长。加大对基础设施、科技创新、生态环保等领域政府投资，推广政府和社会资本合作(PPP)模式，引导和撬动社会资本更多投向制造业、战略性新兴产业和现代服务业。落实企业投资自主权，发挥民间投资主力军作用。支持企业上市，扩大直接融资规模。全年固定资产投资达到4800亿元以上。

优化发展环境促进增长。全面落实降低税率、降低电价、降低社会保险费、降低企业住房公积金缴存比例等相关政策，规范中介服务，清理涉企收费，改善物流服务，严格把握最低工资标准，切实降低企业经营成本。有针对性地帮扶遇到暂时困难的重点企业。强化改进政府服务，优化企业经营环境。

（二）加快产业转型升级

狠抓项目建设不动摇，提高供给能力、调整供给结构、优化供给质量，努力使产品和服务更好地满足市场需求，打造长春经济升级版。

落实“中国制造2025”长春实施纲要。积极推动大众Q工厂、一汽乘用车研发中心、长客动车检修、中粮聚乳酸制品等50个重大产业项目建设。加快建设新能源汽车、通用航空、智能制造等一批新型产业园区。不断壮大先进装备制造、光电信息、生物制造、医药健康、新能源及新能源汽车、新材料等战略性新兴产业。积极推动企业技术改造和产品创新。支持存量企业整合、并购、重组。工业投资增长10%以上，战略性新兴产业投资增长15%以上。

落实率先实现农业现代化总体规划。启动建设5个现代农业实验区。适度调减普通玉米种植面积，积极发展高效特色农作物。强化品牌建设，不断提升长春大米知名度和附加值。加快发展都市休闲农业。综合农机化水平提高2.5个百分点。土地流转比例提高4个百分点。启动实施皓月200万头优质肉牛养加销一体化等一批重大项目。完成免疫无口蹄疫区建设。

落实推进服务业创新发展实施意见。实施服务业发展攻坚，推动服务业发展提速、比重提高、水平提升。加快建设万达、诺睿德、中东广场等商业综合体。规划建设一批特色商业街区。推动大数据、电子商务、服务外包、文化创意、动漫设计等现代服务业集聚区建设。大力发展旅游业。壮大会展业规模。推进共同物流配送。培育现代保险、互联网金融，加快建设东北亚区域性金融服务中心。

（三）全力实现创新驱动

采取更加有力的措施，发展新产业、新业态、新技术、新商业模式，努力营造大众创业、万众创新的生动局面。

培育新兴产业。实施“互联网+”行动计划，推动新一代信息技术与制造业、服务业深度融合。促进制造业企业开展个性化定制、柔性化生产，推动生产性服务业向专业化转变、向价值链高端延伸，支持生活性服务业加快向精细化和高品质提升，构建新兴产业集群。加快“智慧长春”建设。完善通信基础设施。

完善创新载体。启动建设国家级长春新区，在结构调整、科技创新、体制机制等方面先行先试、大胆实践。积极引导国家级开发区强化创新驱动，培育产业集群，加快转型升级。续建长东北科技创新中心、北湖科技园。完善众创、众包等小微企业孵化基地，力争新增创业孵化面积50万平方米。

强化技术创新。支持高校院所建设重点实验室。推进产学研协同创新，实施10个重大科技攻关、10个重大科技成果转化项目。培育10户制造业创新中心、100户企业技术中心。逐步建立鼓励创新创业的收入分配机制。推进“吉林一号”卫星图像数据商业化应用。加快无人机产业化进程。

优化创新环境。加强对中小微企业金融服务，降低创业创新门槛，营造破束缚、汇众智、促创新的良好氛围。强化知识产权管理保护。启动建设人力资源产业园。努力破除人才壁垒，多渠道选用人才，加快建设“人才强市”。全年力争15户企业新三板挂牌，新增50户科技型“小巨人”企业。

（四）深入推进改革开放

围绕供给侧结构性改革，实施一批标志性、引领性、突破性的改革措施，为发展提供持续动力。

继续实施简政放权。严格落实权力清单、责任清单，启动编制负面清单，放管结合、优化服务，激发市场活力和社会创造力。接住用好国家、省下放的审批事项，进一步削减市级行政审批事项。推行行政审批全流程运行管理，完善审批服务。市级行政审批项目全部进入政务大厅，做到大厅之外无审批。

推进重点领域改革。深化投融资、商事制度、市属国企国资等重点领域改革，推动市场在资源配置中起决定性作用。积极开展水生态文明建设、农村金融综合改革、民营经济综合配套改革试点。支持在长央企改革、中省直公立医院综合改革。发展混合所有制经济。推进财税体制改革，建立跨年度预算平衡机制。

扩大开放促进改革。加大招商引资力度，实际利用内、外资分别增长12%和10%左右。不断完善兴隆综合保税区功能。推进“长满欧”综合物流通道建设。开通至珲春、符拉迪沃斯托克（海参崴）的电商货物陆路运输通道。谋划建设东北中韩自贸区示范市。支持一汽、长客等企业开展国际产能合作。

（五）提高城市建管水平

牢固树立人民城市为人民的思想，转变城市发展方式，提高城市承载能力，优化城市环境质量。

构建现代交通体系。续建机场大道、亚泰大街南延长线、吉林大路东延长线、硅谷大街延长线、长吉互通立交桥等重点工程。完善南部新城路网。打通一批断头路、卡脖路。改造一批重要交通节点。建设一批人行过街天桥。加快停车场建设。机动车停车泊位基本做到应划尽划。推进龙嘉机场二期扩建。

深入创建公交都市。地铁1号线试通车，地铁2号线、北湖快轨建设取得实质性进展。更新公交车辆400台。完善南部新城等重点区域公交线网。新建一批公交换乘枢纽、港湾式停靠站。每个城区、国家级开发区各建2处公交首末站。推行公交“一卡通”。完善公交专用道配套设施。

提升综合承载能力。基本完成城区二次供水设施改造。启动新一轮高压电网建设。改造供水管网380公里。新建改造燃气管网210公里。改造供热管网100公里。新增供热能力500万平方米。完成地下综合管廊建设10公里以上，逐步消除“马路拉链”现象。

推动城市管理创新。扎实开展市容环境综合整治。继续改造提升城市出入口。开展城市设计，培育城市风貌。争创国家历史文化名城。创新城市管理体制机制，积极推动管理重心下移，努力提高城市管理人性化、精细化、科学化水平。

（六）全面加强生态保护

把环境保护摆上更加突出的位置，下决心用硬措施完成硬任务，推动生态环境质量不断提升。

强力治理环境污染。淘汰10吨以下燃煤小锅炉500台、黄标车1.3万辆，秸秆综合利用率提高3个百分点。统筹推进伊通河及其支流截污治污和生态修复。实施一批污水处理厂提标改造。治理城区黑臭水体。推动莲花山退耕还林还水还湿工程。

深入实施节能减排。积极调整能源结构，促进煤炭高效利用，扩大供暖煤改气、煤改电规模。实行能源、资源消耗总量和强度控制。深化资源性产品价格改革，居民用气实行阶梯价格。续建餐厨垃圾处理厂、三道垃圾场环保生态公园。

创建国家森林城市。加快建设南溪湿地，启动建设7个公园，新植街路20条，新建大块绿地9块，绿化改造四环路。植树造林3000公顷。绿化美化村屯300个。建设海绵城市，减少硬化铺装，提高雨水自然蓄积能力。

（七）千方百计改善民生

着力践行以人民为中心的发展思想，实施第4个幸福长春行动计划，办一批群众最关心、最直接、最迫切的民生实事。

确保就业稳定。积极扶持高校毕业生、农村转移劳动力、城乡就业困难人员和退役军人就业，开发就业岗位13.5万个，城镇新增就业12万人，农村劳动力转移就业100万人次。对有需求的应届高校毕业生创业培训实现全覆盖。列支1.5亿元失业保险基金，对不裁员、少裁员企业提供稳岗补贴。

帮扶困难群众。城镇和农村低保标准分别提高到上年度人均可支配收入的20%和30%以上。为重度残疾人和贫困残疾人提供护理补贴、生活补贴。实施精准脱贫，确保5.3万名农村贫困人口脱贫。加强贫困村公路建设。优先改造农村贫困家庭危房。

强化养老服务。以政府购买服务方式为城区空巢、失能、失独老人提供居家养老服务。引导社会力量建设多种类型、不同档次的养老机构，新增养老床位3000张。推行高龄老人医疗照护保险、晚期癌症患者舒缓疗护。老年人免费乘坐公交车年龄放宽到65周岁、范围扩大到郊线车。

提升公共服务。继续实施基础教育质量提升工程，推动教育由均衡发展向内涵发展转变。大力发展学前教育、职业教育、民办教育，普及高中段教育。51家公立医院逐步取消药品加成，实行零差率销售。实施普遍二孩政策，强化服务保障支撑。启动少儿图书馆新馆建设。1692个村全部建成村级“一站式”服务群众平台。

改善生活条件。新建续建保障性住房8000套，建设回迁房8456套。实施238万平方米“暖房子”工程。综合改造30个老旧散小区。更新改造部分居民小区陈旧老化地下管网。解决6万农村居民饮水安全问题。主城区周边建成农村公路250公里以上，同步配建公交设施。完善城区“十分钟健身圈”，大力开展全民健身活动。

（八）切实强化社会治理

推动社会治理方式精细化和治理能力现代化，维护群众利益，保障城市安全，促进社会和谐。

积极化解社会矛盾。充分利用信访机构接待、局长接待日、市长公开电话等渠道，掌握群众诉求，解决群众困难。完善人民调解、行政调解、司法调解联动体系。推进幸福社区建设，提高社会组织自治管理水平。强化覆盖城乡的公共法律服务体系建设。

全力维护城市安全。创建国家安全发展示范城市，坚决遏制重特大事故发生。增强地震防灾减灾能力，通过国家防震减灾示范城市验收。创建国家食品安全城市，完善食品安全监管体系，切实保障群众“舌尖上的安全”。强化社会治安综合治理和防控体系建设，严厉打击刑事犯罪，努力提升人民群众的安全感。

深入开展双拥共建活动，推动军民融合深度发展，争创全国双拥模范城“八连冠”。

各位代表！实现“十三五”奋斗目标，完成今年各项工作任务，必须加强政府自身建设。

我们要坚持用创新思维来引领。深入开展解放思想大讨论，用新的思想、思维、思路破解难题，用新的信念、理念、观念谋划发展，用新的办法、举措、行动推动振兴，努力做到变中求新、变中求进、变中突破。

我们要坚持用法治理念来推动。坚持法无授权不可为、法定职责必须为。自觉接受市人大及其常委会的法律监督、工作监督，主动接受市政协民主监督，认真听取民主党派、工商联、无党派人士和各人民团体意见。

我们要坚持用纪律意识来强化。巩固党的群众路线教育实践活动和“三严三实”专题教育成果，始终把纪律和规矩挺在前面，严格执行廉洁自律准则，与一切腐败行为作斗争。厉行节约、反对浪费，严格控制“三公”经费，继续降低行政成本。

我们要坚持用宗旨观念来服务。坚持一切从人民群众根本利益出发，全心全意为人民服务，努力为群众排忧解难。把严格管理和热情关心结合起来，推动广大公务员心情舒畅、充满信心，积极作为、敢于担当。

各位代表！站在新的历史起点、加快推动振兴发展，是760万长春人民的殷切期盼、是我们义不容辞的责任使命。让我们紧密团结在以习近平同志为总书记的党中央周围，在省委、省政府和市委的正确领导下，凝心聚力、锐意进取、扎实工作，不断开拓新局面，持续创造新业绩，为实现“十三五”奋斗目标、全面建成小康社会而努力奋斗！
